# Individual Variations in Maternal Care Early in Life Correlate with Later Life Decision-Making and c-Fos Expression in Prefrontal Subregions of Rats

**DOI:** 10.1371/journal.pone.0037820

**Published:** 2012-05-31

**Authors:** Felisa N. van Hasselt, Leonie de Visser, Jacintha M. Tieskens, Sandra Cornelisse, Annemarie M. Baars, Marla Lavrijsen, Harm J. Krugers, Ruud van den Bos, Marian Joëls

**Affiliations:** 1 SILS-CNS, University of Amsterdam, Amsterdam, The Netherlands; 2 Department of Neuroscience & Pharmacology, Rudolf Magnus Institute, UMC Utrecht, Utrecht, The Netherlands; 3 Department of Animals in Science and Society, Division of Behavioral Neuroscience, Rudolf Magnus Institute, Faculty of Veterinary Medicine, Utrecht University, Utrecht, The Netherlands; Radboud University, The Netherlands

## Abstract

Early life adversity affects hypothalamus-pituitary-adrenal axis activity, alters cognitive functioning and in humans is thought to increase the vulnerability to psychopathology–e.g. depression, anxiety and schizophrenia- later in life. Here we investigated whether subtle natural variations among *individual* rat pups in the amount of maternal care received, i.e. differences in the amount of licking and grooming (LG), correlate with anxiety and prefrontal cortex-dependent behavior in young adulthood. Therefore, we examined the correlation between LG received during the first postnatal week and later behavior in the elevated plus maze and in decision-making processes using a rodent version of the Iowa Gambling Task (rIGT). In our cohort of male and female animals a high degree of LG correlated with less anxiety in the elevated plus maze and more advantageous choices during the last 10 trials of the rIGT. In tissue collected 2 hrs after completion of the task, the correlation between LG and c-fos expression (a marker of neuronal activity) was established in structures important for IGT performance. Negative correlations existed between rIGT performance and c-fos expression in the lateral orbitofrontal cortex, prelimbic cortex, infralimbic cortex and insular cortex. The insular cortex correlations between c-fos expression and decision-making performance depended on LG background; this was also true for the lateral orbitofrontal cortex in female rats. Dendritic complexity of insular or infralimbic pyramidal neurons did not or weakly correlate with LG background. We conclude that natural variations in maternal care received by pups may significantly contribute to later-life decision-making and activity of underlying brain structures.

## Introduction

Psychiatric diseases such as schizophrenia, anxiety disorders and depression involve disruptions of higher executive processes, which are under control of the prefrontal cortex (PFC) [1], [2]. Exposure to stressful events is -in vulnerable individuals- an important risk factor for the development of these diseases and thought to affect the function and dendritic structure of the prefrontal cortex [3], [4], [5], [6], [7], [8]. Interestingly, regulation of the hypothalamo-pituitary-adrenal (HPA) axis is partly controlled by the PFC and disrupted in many psychiatric disorders [9], [10], [11] which suggests an iterative loop in which hormones secreted after HPA activation may further exacerbate dysfunction of the PFC.

The risk of developing psychopathology is particularly increased after early life adversity [12], [13], [14], [15]. The presumed influence of early life adversity on brain structure and neurotransmission can be addressed in detail in appropriate animal models. Indeed, some studies show that interfering with mother-pup interactions affects the development and function of the PFC. For instance, repeated neonatal maternal separation (MS) in rodents, a frequently used model for adverse early life experience, was reported to alter dendritic morphology and spine density in PFC pyramidal neurons [16], [17], [18].

Studies examining natural variations in maternal care during the early postnatal period reported that maternal licking and grooming (LG) of the pups affects neuroendocrine responses, cognitive performance and neuronal development. Pups from mothers providing very low amounts of LG (Low-LG) to their entire litter showed -in adulthood- enhanced HPA axis responses [19], increased anxiety [20] and emotional memories [21], impaired spatial memory performance [22] and reduced hippocampal dendritic complexity [21], [23], when compared to pups from mothers that spent a very high amount of time licking and grooming (High-LG) their litter.

Since early life experiences and alterations in prefrontal cortex function are relevant for psychopathology, we here examined whether variations in the amount of maternal care also correlate with decision making processes – which critically depend on an intact PFC [24] – as well as the associated neuronal activation and dendritic complexity in the PFC. Earlier studies on the impact of maternal care for behavior and neuronal development focused on entire litters from High-LG and Low-LG mothers [19], [20], [21], [22], [23]. However, there is considerable within-litter variation [25], [26], [27], [28]. In the current study we therefore investigated whether the amount of care that *individual* pups within a litter receive from their mother correlates with PFC behavior and function. This approach enables to directly study the relationship between maternal care and PFC-dependent behavioral processes, activation and PFC structure, with a minimal role of confounding genetic factors.

We first tested all animals for their behavior in an elevated plus maze, since this parameter was earlier found to predict later performance in a decision-making task. Next, the relationship between LG received by individual pups early in life and PFC-dependent decision-making performance in adulthood was examined in a rodent version of the Iowa Gambling Task (rIGT, [29]). We also assessed the underlying neural circuitry using the immediate early gene c-fos as a marker for neural activity in structures associated with IGT performance, i.e. prefrontal, striatal and limbic areas (see [30]). Finally, we tested putative LG-related differences in dendritic morphology of two prefrontal cortex subregions of interest, i.e. the infralimbic and insular cortex.

## Materials and Methods

### Maternal care

Male and female outbred Long Evans rats were purchased from Harlan (Indianapolis, US) at approximately 2.5 months of age and allowed to habituate to the animal facility in Amsterdam. Then, two females were housed with one male for one week to allow mating, and after another week of paired-housing, the females were placed separately in large observation cages (30 cm×55 cm×45 cm). Maternal care observations commenced on postnatal day 1 (PND1; with PND0 being the day of birth), after culling the litters by randomly selecting eight healthy-looking pups (preferably four males and four females). The observation procedure has been extensively described by Champagne et al. (2003) [31] and was comparable to what we used in our earlier studies [26], [27]. Briefly, maternal behavior was scored every three minutes during five one-hour observation sessions daily (7:00, 10:00, 13:00, 17:00 and 20:00 hrs) for seven days, resulting in a total of 700 observations for each litter. Maternal care observations are generally done during the light period because dams show less maternal care during the dark period; the amount of maternal care is evenly distributed over the light period [31]. Several specific maternal behaviors were scored, including licking and grooming (LG), particularly towards individual pups within each litter. In order to be able to identify the pup that underwent licking and grooming, all pups were uniquely marked every morning until weaning with a non-scenting, non-toxic surgical marker (Codman, Johnson & Johnson, Brunswick, NY). We were able to distinguish which pup was being licked and groomed in about 60% of the cases, and since this percentage varied slightly between litters, we corrected for this with the following equation: (% individual LG observed)/(% total LG identified) * 100%. The handling of the pups which is inevitably associated with marking their fur might elicit an increase in overall licking and grooming [19], [32]. However, both a pilot study carried out previously in our lab (C.A. Oomen, M. Joëls, unpublished data) and earlier observations by Champagne et al. (2003) did not show any differences in maternal licking and grooming behavior in response to pup marking. In addition, one could argue that even if the amount of maternal care towards the whole litter was somewhat shifted due to the experimental procedures, within-litter differences in pup-preference should not be affected.

The animals were kept on a 12 h light/dark schedule (lights on at 8:00 hrs) until PND24, when they were weaned and ear-punched. Then they were moved to a different room where the light cycle was reversed (lights on at 21:00 hrs), so that eventually they could be tested in the dark phase, when they are active. During the entire experiment, temperature and humidity were maintained at 20–22°C and 40–60% respectively, and food and water were available *ad libitum*, unless otherwise specified. Post-weaning, the rats were group-housed with same-sex non-littermates.

All rats were first exposed on PND35 to a protocol which determined the effect of differences in %LG received on play behavior, of which the results have been published elsewhere [28]. The latter manuscript also contains data on the within-litter variation of this cohort of rats (six litters, PND1–PND7: Figure 1 in [28]). More specifically, %LG received varied considerably between pups within litters (overall range 0.00% to 2.30% LG, n = 48). In general, males received significantly more LG than females [28], which has been described before and has been explained by the fact that male pups require more LG to be able to urinate and defecate than female pups (i.e. [33]). We cannot exclude that pups that are larger and stronger at birth can maneuver themselves easier and closer to their mother and thus receive more LG. Although we have no information on birth weights of our pups, we did determine weaning weights and did not find a correlation with %LG in this cohort of rats (data not shown).

**Figure 1:**
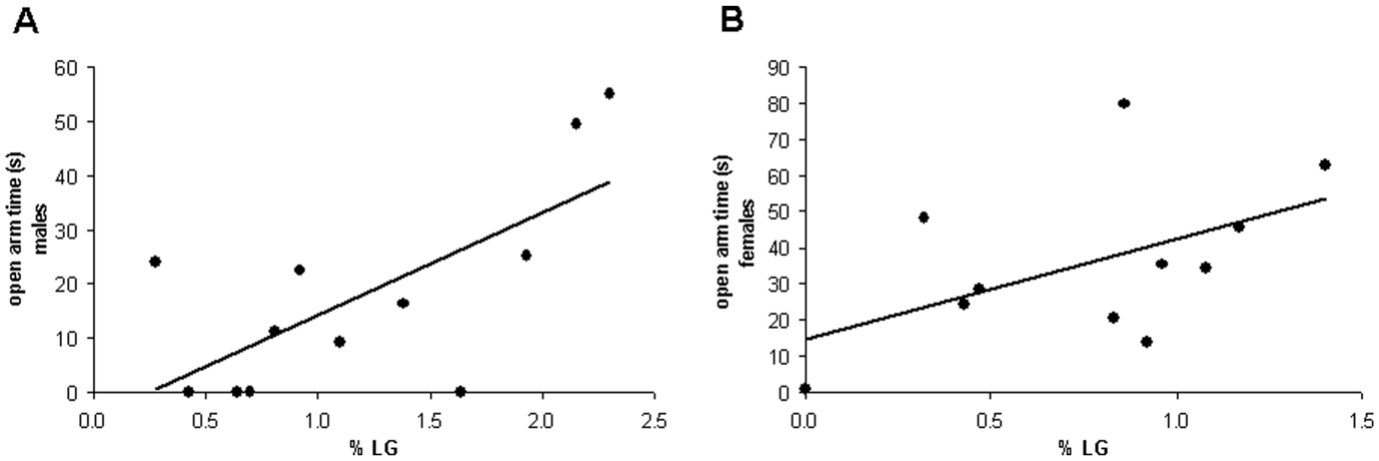
Elevated Plus Maze. (A) In ten-week-old male offspring a positive correlation emerged between %LG and open arm time on the Elevated Plus Maze (n = 12, r = 0.690, p = 0.013), indicating reduced anxiety in animals with higher LG scores. (B) Similarly, in 8-week-old females, there was a positive trend between these parameters (n = 11, r = 0.511, p = 0.108).

A subset of rats (n = 24, 12 males/12 females) entered the decision-making experiment at around eight (females; PND56) to ten (males; PND70) weeks of age. Because the animals used were from the same breeding cohort, they were all born around the same time. Yet, only 12 animals can be tested in the rIGT concurrently. Instead of using a wide age-range for both sexes and also because it is known that both male and female behavior is affected by the presence and smell of the opposite sex, we decided to test males and females separately. Females were tested at a slightly younger age because they mature faster and thus can be considered to be at a comparable level of maturity at PND56 as males of a slightly later age.

Another group of 19 animals (10 males/9 females) were used to assess prefrontal dendritic morphology (ten weeks of age; PND70). All experimental procedures used in this study were approved by the animal ethical and welfare committee of the University of Amsterdam.

### Elevated Plus Maze (EPM)

All 24 animals that were designated to participate in the decision-making experiment (see below) were first tested on the EPM. The maze was made of black PVC, consisted of four arms (50×10 cm) – two open and two enclosed by 30 cm high walls, forming a cross with the center area – and was elevated 60 cm above the ground. Animals were tested during the dark phase of their circadian light cycle, though under bright light conditions, to amplify putative maternal care related differences in anxiety level. Each rat was put on the center platform facing one of the open arms and allowed to freely explore the maze for 5 minutes. Between trials the maze was cleaned with ethanol and water, and dried thoroughly with clean paper towels. All test sessions were recorded for later analysis of spatiotemporal measures (i.e. the time spent in the open and closed arms and the number of entries into each arm) using the Observer 5.0 software (Noldus Information Technology B.V., Wageningen, The Netherlands).

### Rodent Iowa Gambling Task (rIGT)

24 animals (12M/12F) along the entire range of LG scores (between 0.32% and 2.30% LG for males; between 0.00% and 1.40% LG for females) were randomly selected for testing in the rIGT. The same apparatus and procedure as previously described was used [29], [30], [34], [35], [36]. Before the start of the experiment, rats were habituated to the test apparatus in a 10 min free exploration trial. Two days later, they were mildly food restricted (approximately 95% of free feeding body weight) and tested during two 5-day periods. On weekend days food was available *ad libitum*. All testing occurred in red-light conditions during the dark phase of the day-night cycle, between 11:00 and 16:00 hrs.

The test apparatus was made of grey PVC and consisted of a start box, choice area and four arms. A trial started by lifting the slide door of the start box, allowing the rat to freely enter the choice area of the apparatus and choose one of the four arms. The chosen arm was closed when the rat had entered that arm with its full body, including its tail. At the end of each arm, rats could either obtain sucrose pellets or quinine-treated sucrose pellets (baited arms; see below) or no pellets at all (empty arms). Each trial had a maximum duration of 6 min, the inter-trial interval was 30 s, and each animal received a total of 120 trials (10 trials during the first 6 days; 15 trials on the final 4 days). Sucrose pellets (45 mg; Bio-Serv, Frenchtown, NJ, USA) - to which rats were familiarized prior to the start of the experiment - were used as a reward while quinine-treated sucrose pellets, that were unpalatable but not uneatable, were used as punishment. There was no significant correlation between the amount of maternal care received early in life and the speed of sugar-pellet consumption during the habituation phase (n = 19, r = −0.39, p = 0.1), indicating no LG-related anhedonic predisposition. As mentioned before, two of the four arms in the maze were empty. These were included to measure non-reward related exploration [29], [30], [34], [35], [36]. The two baited arms consisted of a ‘bad’ arm and a ‘good’ arm. In the ‘bad’ arm, the rats received occasional big rewards (three sucrose pellets in 1 out of 10 trials) among frequent punishments (three quinine-treated sucrose pellets in 9 out of 10 trials). In the ‘good’ arm, the rats received frequent small rewards (one sucrose pellet in 8 out of 10 trials) and infrequent punishments (one quinine-treated sucrose pellet in 2 out of 10 trials). This provided the same principle as in the human IGT: an option with a chance of a big reward (3 sucrose pellets), but with little long-term success (3 sucrose pellets per 10 trials; cf. decks A and B; [37]) and an option with a chance of a small reward (1 sucrose pellet), but with bigger long-term success (8 sucrose pellets per 10 trials; cf. decks C and D). The location of the baited and empty arms, as well as ‘good’ and ‘bad’ arms was counterbalanced across subjects.

To determine the performance of the rats, the number of choices for the most advantageous option were calculated and expressed as a fraction of the total number of trials per block. Choices were calculated in blocks of 10 trials. Scores during the last block of 10 trials (trial 111–120) were taken as a measure of final rIGT performance. The number of sucrose pellets collected during the entire task (trial 1–120) was used as a measure of overall task performance to reflect the final “budget” (cf. monetary budget in the human IGT, [38]. In addition we determined the total number of visits to either of the empty arms and the number of switches between different arms to assess exploratory behavior [29], [30], [34], [35], [36]. Finally, we measured win-stay/lose-shift behavior after encounters with the sucrose reward (win) or the quinine punishment (lose) in the advantageous arm. Win-stay and lose-shift were corrected for the total number of encounters with sugar and quinine respectively [30].

### C-fos immunohistochemistry

Two hours after the last trial in the rIGT, the animals were rapidly decapitated. Their brains were removed, immediately snap-frozen on dry-ice and stored at −80°C. Coronal sections (20 µm) were cut on a cryostat (Leica CM3050S), mounted on Starfrost adhesive slides (Knittel Glaser, Waldemar Knittel, Germany) and stored at −20°C. For the immuno-histochemical detection of c-fos (at protein level), rabbit anti-c-fos (Calbiochem, Darmstadt, Germany) was used. During the staining procedure the sections were rinsed several times after every step in 0.01 M phosphate-buffered saline (PBS; pH 7.4). First, the sections were dehydrated. Endogenous peroxidase was blocked by treatment with H_2_O_2_ (0.1%) for 30 min. Sections were pre-incubated with 5% normal donkey serum (NDS) and 1% bovine serum albumin (BSA) in PBS (PBS-BSA 1%+NDS 5%) for 30 min before the rabbit anti-c-fos incubation (1∶4000 in PBS-BSA 1%+NDS 5%, 4°C, 24 h). Negative controls were incubated with the PBS-BSA 1%+NDS 5% solution. Next, the sections were incubated with donkey–anti-rabbit IgG Biotin SP conjugate (1∶400 in PBS-BSA 1%+NDS 5%, Jackson ImmunoResearch Laboratories, Inc., PA, USA) for 45 min. Subsequently, the sections were incubated with avidin-horseradish peroxidase solution (1∶400 in PBS-BSA 1%+NDS 5% VECTASTAIN® ELITE ABC, Brunswich Chemie, Amsterdam, The Netherlands) for 60 min. Then, slices were pre-incubated with inactive diaminobenzidine tetrahydrochloride (DAB, Sigma-Aldrich, St. Louis, MO, USA) solution containing nickel sulphate. To activate DAB for visualization of bound peroxidase complexes, the substrate H_2_O_2_ (30%, 1∶2000) was added to the DAB solution and incubated for 5 min. Afterwards the sections were dehydrated in alcohol and coverslipped.

The images of brain sections were projected (10× magnification) and digitized using an Olympus BX 51 microscope (Olympus, Tokyo, Japan) with a high-resolution digital camera interfaced with a computer. The anatomical localization was aided by use of adjacent Nissl stained sections and illustrations in a stereotaxic atlas [39]. The following brain regions were investigated: orbitofrontal cortex (OFC; +4.20 from bregma), insular cortex (insular; +1.92 from bregma), medial prefrontal cortex, i.e. cingulate cortex (Cg1; +2.52 from bregma), prelimbic (PrL; +2.52 from bregma) and infralimbic cortex (IL; +2.52 from bregma), dorsolateral striatum (DLS; +1.92 from bregma), dorsomedial striatum (DMS; +1.92 from bregma), nucleus accumbens core (NaC; +1.92 from bregma), nucleus accumbens shell (NaS; 1.92 from bregma), basolateral amygdala (BLA; −2.52 from bregma), central nucleus of the amygdala (CeN; −2.52 from bregma), dentate gyrus (DG; −3.36 from bregma) and CA1 regio of the hippocampus (CA1; −3.36 from bregma). For each region at least two overt landmarks were used. For quantitative analysis of c-fos positive cells, the program Leica QWIN (image processing and analysis software, Cambridge, UK) was used. Only right hemispheres were analyzed, using two subsequent sections per animal. For all regions of interest, the number of positive cells was then averaged for each animal and expressed per mm^2^.

### Morphology

For further morphological survey we selected two areas which exhibited clear c-fos immunoreactivity related to 1) rIGT performance or 2) LG background, i.e. the infralimbic cortex and the insular cortex respectively. To determine dendritic complexity of principal neurons in these areas, we used the Golgi-Cox method, as described previously [27], [40]. Ten males and 9 females were sacrificed at around 2.5 months of age, concurrent with the animals that were tested in the rIGT. We rapidly removed their brains and immediately put one of the hemispheres (right) in a vial containing Golgi-Cox solution (1% potassium dichromate, 1% mercuric chloride, 0.8% potassium chromate). The tissue remained immersed in this solution in the dark for 28 days, and was subsequently dehydrated and embedded in celloidine. The forebrain was cut in 200 µm thick slices using a vibratome, sections were stained as described by Boekhoorn et al. (2006) and mounted on glass slides.

For each animal, four cells that were randomly chosen from different slices were imaged and traced using ImagePro and NeuroDraw software. Only cells that *i)* were thoroughly filled, *ii)* were located in cortical layer 2/3, *iii)* had their soma in the middle plane of the slice, and *iv)* did not substantially interfere with neighboring cells or debris, were selected for analysis. Dendritic tracing was carried out by an experimenter blind to the background of the animals and several morphological parameters were analyzed, including total dendritic length, average branch length, number of branch points, and dendritic complexity index [DCI = (Σ branchtip orders+# of branch tips)/(# of primary dendrites) * (total dendritic length)].

### Statistical analysis

Statistical analyses were conducted using SPSS 11.0 for Windows. All correlations were tested using linear regression with %LG as the independent (predictor) variable. Male and female data were only pooled for a certain correlation if *i)* the direction of that correlation was similar in both sexes and if *ii)* neither of the parameters in the correlation (e.g. %LG, IGT performance) differed significantly between sexes (analyzed by an independent Student's t-test). To determine the effect of maternal care on the relationship between rIGT performance and c-fos staining we used partial correlations, controlling for %LG. Furthermore, we examined whether differences existed between the learning curves of animals with different LG backgrounds using a split-half approach. Animals were divided into two equally sized groups: 12 animals with the lowest LG scores (5M/7F) and 12 animals with the highest LG scores (7M/5F). The number of advantageous choices was calculated in blocks of 10 trials and we used a repeated measures ANOVA to assess task progression in both groups (within-subjects factor: trial block; between-subjects factor: LG group).

## Results

### Elevated Plus Maze

All animals selected for the rIGT were first tested on the Elevated Plus Maze. The average time spent on the open arms of the maze differed between sexes (mean ± SEM: 17.71±5.42 sec in males versus 35.74±6.78 sec in females; t = 2.095, df = 21, p = 0.048) and therefore we analyzed them separately. In males, a significant positive correlation emerged between %LG and time spent on the open arms (n = 12, r = 0.690, p = 0.013; Figure 1A), whereas in females this was only a positive trend (n = 11, r = 0.511, p = 0.108; Figure 1B). Individual %LG scores did not correlate with the number of closed arm entries, neither in males (n = 12, r = 0.137, p = 0.672) nor in females (n = 12, r = −0.069, p = 0.830), suggesting that there was no difference in general activity between animals.

### Rat Iowa Gambling Task (rIGT)

After testing animals in the Elevated Plus Maze they were studied at least 1 week later in the rIGT. Male and female rats did not differ in average rIGT performance (fraction of advantageous choices; mean ± SEM: 0.58±0.06, n = 12, for males and 0.58±0.07, n = 12 for females; t = 0.000, df = 22, p = 1.000). In addition, gonadal hormone status was previously shown not to affect decision-making in the IGT, neither in rats nor in humans [36], [41]. Therefore we pooled male and female data for further correlational analysis.

**Figure 2:**
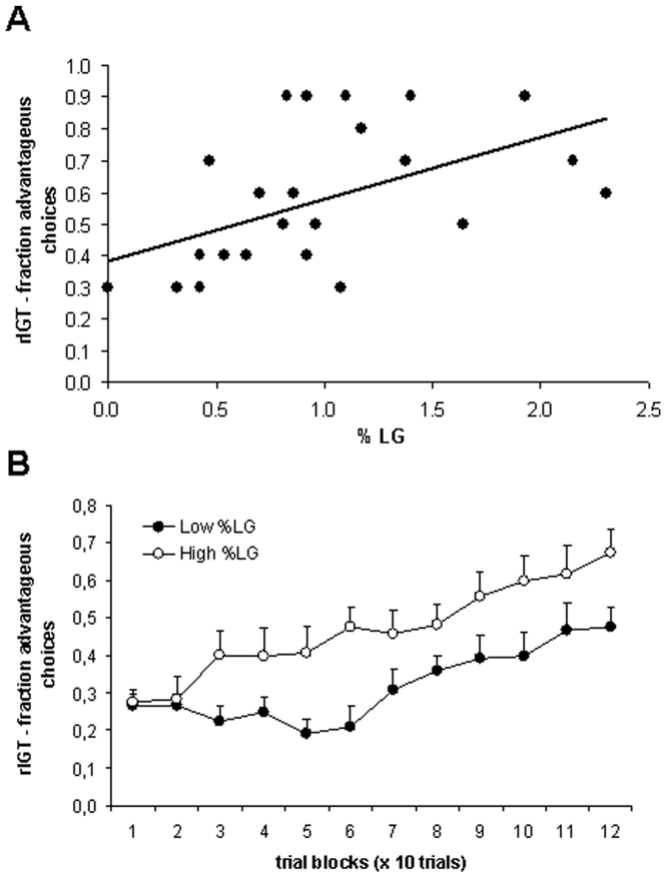
Performance on the rodent Iowa Gambling Task. (A) In young-adult males and females (approx. 3 months old) there was a positive correlation between task performance on the rIGT, as indicated by the number of advantageous choices made during the last block of 10 trials, and %LG (n = 24, r = 0.521, p = 0.009). Thus, animals with higher LG scores perform better on a higher cognitive decision-making task. (B) When dividing the experimental group in ‘Low’ and ‘High’ LG animals by median split, a significant interaction effect between %LG and trial block emerged (10 trials per block; repeated measures ANOVA, main effect of group: F(1,22) = 6.485, p = 0.018; LG-group * trial block interaction: F_(11,242)_ = 1.807, p = 0.05; trial block: F_(11,242)_ = 14.544, p = 0.001). This suggests delayed learning in the ‘Low’ LG group, particularly in the first part of the task.

We observed a highly significant positive correlation between the %LG and the number of advantageous choices made in the rIGT during the last block of 10 trials (n = 24, r = 0.521, p = 0.009; Figure 2A). To examine task progression, a median split into ‘Low’ and ‘High’ LG animals was applied. This yielded a significant difference in improving performance across trial blocks between the two subgroups (repeated measures ANOVA, trial block, F_(11, 242)_ = 14.544, p = 0.001; LG group * trial block interaction, F_(11,242)_ = 1.807, p = 0.05; Figure 2B): rats that received relatively high maternal care showed a steady increase in the number of choices for the advantageous option from trials 11–20 onwards, while rats that received less maternal care tended to show a decrease in choosing for the advantageous option in the first trial blocks, and only showed a steady increase after trials 61–70. These differences in choice behavior between animals with high and low %LG were accompanied by a significant positive correlation between %LG and the total number of sugar pellets earned during the task (n = 24, r = 0.420, p = 0.02), indicating that rats that received more maternal care had a higher overall yield. A significant negative correlation existed between %LG and the number of visits to the empty arms during the task (n = 24, r = −0.421, p = 0.04), which points to increased non-reward related exploratory behavior in animals with lower LG scores. The total number of switches between arms did not correlate with %LG (n = 24, r = 0.017, p = 0.938). A positive trend was found for the relationship between %LG and win-stay behavior across all 120 trials (n = 24, r = 0.366, p = 0.08), while no significant effect or trend was observed for lose-shift behavior (n = 24, r = −0.132, p = 0.537). We did not observe a correlation between anxiety, as measured by open arm time on the Elevated Plus Maze, and rIGT performance measured in the last block of 10 trials (n = 21, r = 0.138, p = 0.551).

### C-fos immunohistochemistry

Significant gender differences in levels of c-fos expression were found in the lateral orbitofrontal cortex (OFC; t = −2.444; df = 22; p = 0.027) but not in any other brain area. Therefore, we only refrained from pooling the male and female data for the lateral OFC.

**Figure 3:**
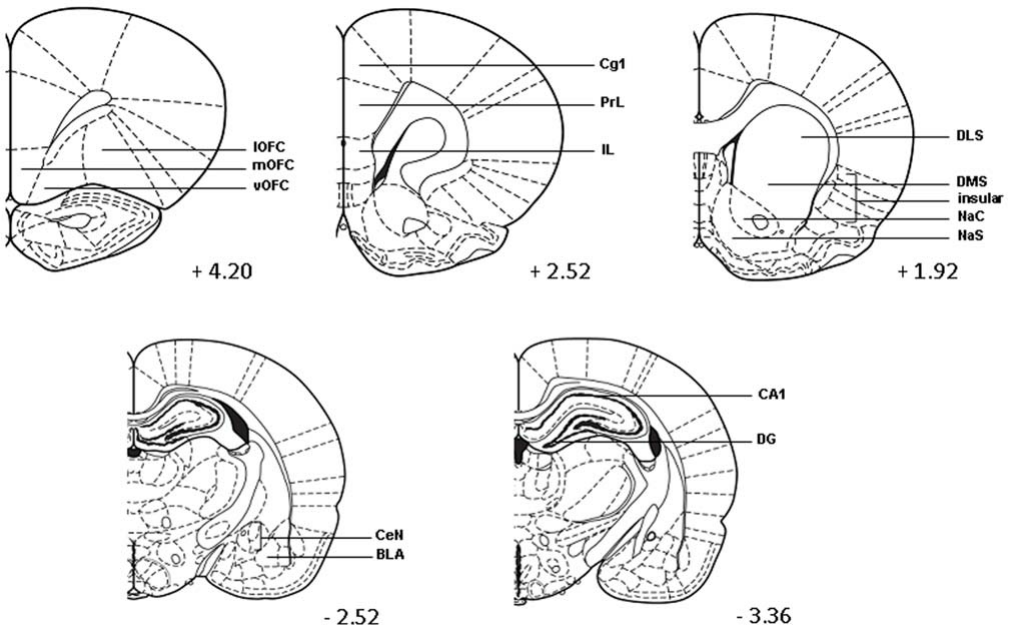
Anatomical localization of brain regions used for analysis of c-fos expression. Anterior-posterior coordinates relative to bregma are stated below each coronal section. Abbreviations: mOFC = medial orbital frontal cortex (OFC)), vOFC = ventral OFC, lOFC = lateral OFC, insular = insular cortex, Cg1 = cingulate cortex, PrL = prelimbic cortex, IL = infralimbic cortex, DLS = dorsolateral striatum, DMS = dorsomedial striatum, NaC = nucleus accumbens core, NaS = nucleus accumbens shell, BLA = basolateral amygdala, CeN = central nucleus of the amygdala, DG = dentate gyrus and CA1 = CA1 regio of the hippocampus. Based on Paxinos and Watson (2005).

First the correlation between rIGT performance and c-fos expression was established in a range of brain areas important for decision making (Table 1; Figure 3). The rIGT performance, expressed as the fraction of advantageous choices during the last block of 10 trials, showed a significant negative correlation with c-fos expression in the mPFC (prelimbic and infralimbic), the insular cortex and the lateral OFC (in females only; males: n = 12, r = −0.018, p = 0.952). There was a negative trend between rIGT performance and c-fos expression in both the cingulate cortex and nucleus accumbens shell. Next, correlations were calculated for %LG and c-fos expression after rIGT performance. Significant negative correlations were found between %LG received early in life and c-fos expression levels in the insular cortex and nucleus accumbens shell. There was a negative trend between %LG and c-fos expression in the lateral OFC (in females only; males: n = 12, r = −0.070, 0.828) and cingulate cortex, and a positive trend in the basolateral amygdala. Finally, partial correlations were calculated between rIGT and c-fos, controlling for %LG, to determine the effect of maternal care on the relationship between rIGT performance and c-fos staining. Table 2 shows that after controlling for %LG, the correlations between rIGT performance and c-fos staining in the cingulate cortex and medial PFC (both prelimbic and infralimbic cortex) became stronger, whereas correlations (significant or trend) between rIGT performance and c-fos expression in the lateral OFC, insular cortex and nucleus accumbens shell were now non-significant (p>0.1). This indicates that maternal care significantly contributes to the relationships between rIGT performance and c-fos labeling in the lateral OFC, insular cortex and nucleus accumbens shell.

**Table 1:** Correlations of c-fos expression with rIGT performance and individual %LG.

Brain area	rIGT-advantageous choices		LG%	
	r =	p =	r =	p =
*Cortical*				
Medial OFC	−.201	.346	−.299	.156
Ventral OFC	−.292	.167	−.330	.115
Lateral OFC (females only)	**−.600**	.039*	−.554	.062^†^
Cingulate (Cg1)	−.369	.076^†^	−.360	.084^†^
Prelimbic (PrL)	**−.418**	.042*	−.344	.100
Infralimbic (IL)	**−.460**	.024*	−.251	.236
Insular	**−.456**	.025*	**−.531**	.008*
*Striatal*				
Dorsolateral striatum (DLS)	−.222	.308	.136	.536
Dorsomedial striatum (DMS)	−.332	.141	−.273	.214
Nucleus accumbens core (NaC)	−.308	.153	−.269	.214
Nucleus accumbens shell (NaS)	−.373	.080^†^	**−.464**	.026*
*Limbic*				
Amygdala central nucleus (CeN)	.148	.500	.222	.309
Basolateral amygdala (BLA)	−.075	.735	.366	.086^†^
Dentate gyrus (DG)	.151	.493	−.096	.663
CA1 region (CA1)	.015	.944	−.251	.236

Correlations between c-fos expression and both rIGT performance and individual %LG (* p<0.05; ^†^ p<0.1). Male and female data were pooled (n = 24), except for the lateral OFC (n = 12).

**Table 2:** c-fos expression and rIGT performance when corrected for %LG.

Brain area	rIGT-advantageous choices controlled for %LG	
	r =	p =
*Cortical*		
Medial OFC	−.247	.395
Ventral OFC	−.419	.135
Lateral OFC (females only)	−.395	.230
Cingulate (Cg1)	**−.552**	.041*
Prelimbic (PrL)	**−.583**	.029*
Infralimbic (IL)	**−.675**	.008*
Insular	−.470	.090^†^
*Striatal*		
Dorsolateral striatum (DLS)	−.229	.430
Dorsomedial striatum (DMS)	−.253	.384
Nucleus accumbens core (NaC)	−.430	.124
Nucleus accumbens shell (NaS)	−.262	.366
*Limbic*		
Amygdala central nucleus (CeN)	.068	.817
Basolateral amygdala (BLA)	−.118	.688
Dentate gyrus (DG)	.280	.332
CA1 region (CA1)	.197	.500

Partial correlations between c-fos expression and rIGT performance, corrected for the effect of LG (* p<0.05; ^†^ p<0.1). Male and female data were pooled (n = 24), except for the lateral OFC (n = 12).

### Morphology

**Figure 4:**
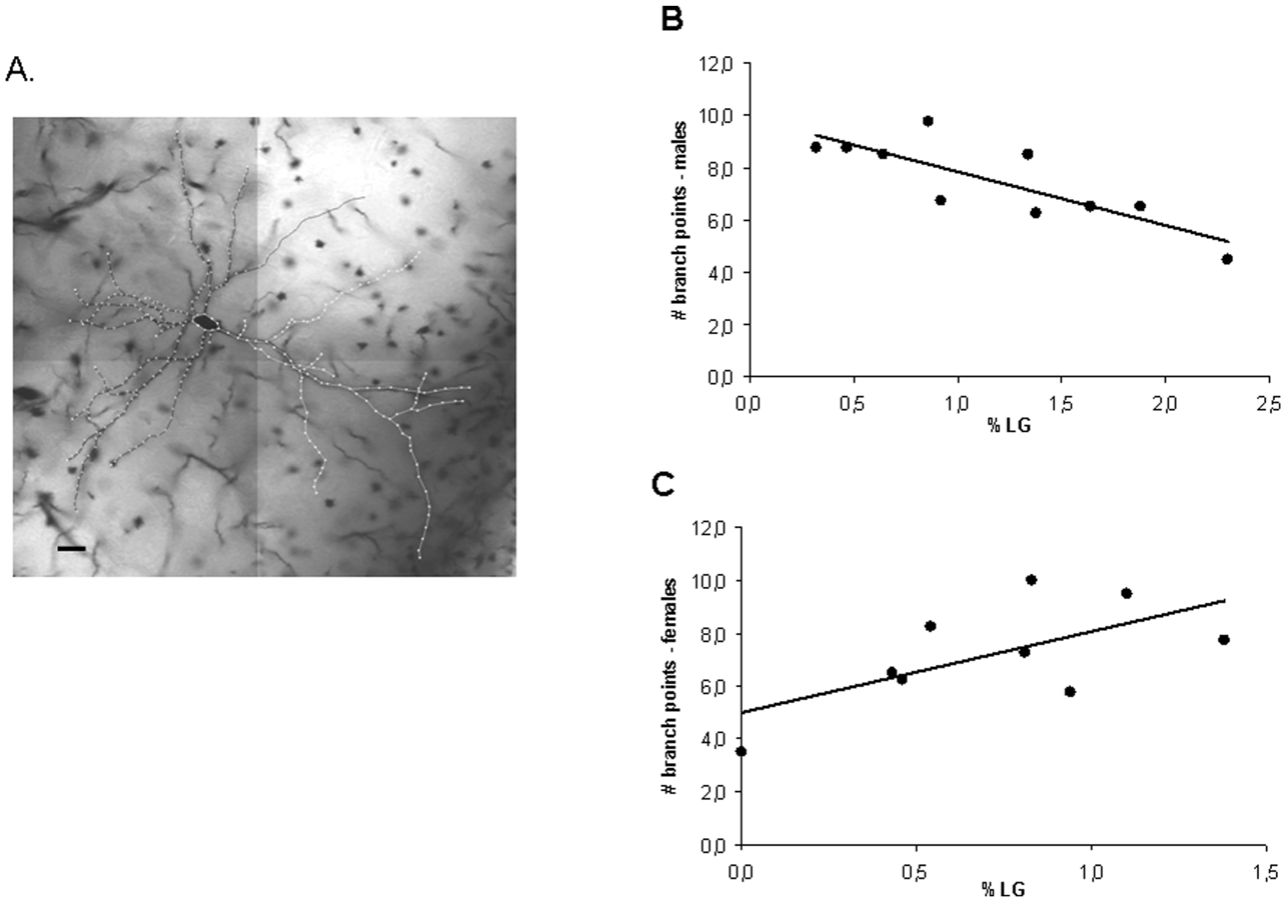
Dendritic morphology in 2.5 month old naïve animals. (A) Typical example of a Golgi-stained pyramidal neuron in the insula and the corresponding dendritic reconstruction drawing. Calibration bar: 20 µm (B) In male infralimbic cortex, %LG correlated negatively and significantly with the number of branch-points in the basal dendrite (n = 10, r = −0.820, p = 0.004). (C) In females, this correlation was positive and just missed significance (n = 9, r = 0.635, p = 0.066).

We next examined if individual differences in maternal care correlate with dendritic morphology in the forebrain. We selected one area which exhibited clear c-fos immunoreactivity related to rIGT performance (infralimbic cortex) and another area for which we observed a correlation with LG background (insular cortex; see typical example of stained neuron in Figure 4A).

Since the direction of the correlation coefficients generally differed between sexes we analyzed males and females separately. In the insular cortex no significant correlations appeared between %LG and pyramidal cell morphology, neither in males nor in females: both for the apical and the basal dendritic tree none of the parameters was affected by individual differences in maternal care (see Table 3). In the infralimbic part of the medial prefrontal cortex the effect of LG on dendritic morphology was different for males and females. In males, the number of branch points (n = 10, r = −0.820, p = 0.004; Figure 4B) in the basal tree showed a significant negative correlation with %LG. The total dendritic length (n = 10, r = −0.575, p = 0.08) and complexity of the basal dendritic tree as measured by the DCI (dendritic complexity index; n = 10, r = −0.557, p = 0.09) only showed a negative trend. No effects of maternal care on the apical dendritic tree were found. In general, these data suggest a more complex basal but not apical dendritic tree in male animals that received lower amounts of licking and grooming. In females, early life background correlated only marginally with morphological properties (Table 3): The correlation between maternal care and the number of branch points showed a positive trend (n = 9, r = 0.635, p = 0.066; Figure 4C), but all other correlations were not significant (see Table 3).

**Table 3:** Dendritic morphology in 2.5 month old naïve animals.

Males				
Parameter	Infralimbic cortex		Insula	
	r =	p =	r =	p =
Apical dendritic length	−.034	.925	−.052	.887
Apical # branch points	−.132	.716	−.058	.874
Apical DCI	−.088	.808	−.168	.642
Basal dendritic length	−.575	.082^†^	.214	.552
Basal # branch points	**−.820**	.004^*^	.032	.929
Basal DCI	−.557	.094^†^	.091	.803

Females				
Parameter	Infralimbic cortex	Insula		
	r =	p =	r =	p =
Apical dendritic length	.456	.217	−.084	.830
Apical # branch points	.523	.149	.041	.917
Apical DCI	.428	.250	.084	.830
Basal dendritic length	.246	.524	−.010	.979
Basal # branch points	.635	.066^†^	.006	.989
Basal DCI	.457	.216	.191	.622

Correlations between individual %LG and dendritic morphology in two forebrain areas, i.e. the infralimbic cortex and the insular cortex, in 2.5 months old naïve animals (* p<0.05; ^†^ p<0.1). Males (n = 10) and females (n = 9) were analyzed separately.

## Discussion

In the present study, we investigated the effects of variations in %LG received by rats during the first postnatal week on decision-making and associated neuronal activity and structure in young adulthood.

### Maternal care model

To address this question we first established the amount of care that *individual* pups within a litter receive from their mother (within-litter model) and used this as an index for correlations with behavior later in life. This approach differs from most studies (reviewed in [42]) which compare the entire offspring from dams that spend a relatively low amount of time LG (>1SD below the mean) with litters from dams that spend a relatively high amount of time LG (>1SD above the mean). The latter (between-litter) model focuses on the extremes of the normal distribution in maternal care, excluding approximately 70% of the litters, which receive moderate amounts of care. Moreover, by comparing entire litters one cannot exclude the contribution of genetic background unless this is ruled out by dedicated cross-fostering experiments. By examining animals over the entire range of LG and using the %LG that was received (regardless of the dam) as a predictor, the influence of these two potential drawbacks is minimized.

The %LG received early in life correlated negatively with the levels of anxiety as measured on the Elevated Plus Maze, which agrees with earlier findings using the more extreme between-litter maternal care model [43]. This effect appeared to be stronger for male than female rats, but it should be noted that in female rats the dwell-time in the open arm time on the Elevated Plus Maze may also reflect exploratory behavior [44]. The differences in EPM behavior between males and females should therefore be cautiously interpreted. Regardless, the findings support that, even within a litter, variations in maternal care can be reliably scored and correlated with anxiety related behavior for male and female offspring.

### Behavioral performance in the rIGT

The correlational analysis indicated that rats receiving low amounts of LG as pup show poorer decision-making -as evident from a lower number of advantageous choices in the rIGT- than rats which receive high amounts of LG. In fact, the performance of some of the rats with a low LG background was hardly above chance level after 120 trials. The poor performance was associated with elevated non-reward related exploration, reflected by a higher number of choices for the empty arms, and a trend towards less win-stay behavior in the advantageous arm. We did not observe a correlation between the time rats needed to consume two sucrose pellets during familiarization and %LG received or the number of advantageous choices in the IGT (data not shown), suggesting that the association between %LG received and rIGT performance is not simply due to differences in sucrose sensitivity. Overall, these findings suggest a choice strategy that is still in an exploratory phase in rats that received little LG, as opposed to rats that received more LG which successfully changed their choice strategy from exploration to exploitation of the most advantageous option.

Earlier studies showed that high anxiety is related to impaired decision-making in the IGT, both in humans [45], [46] and in rats [30]. Although we did not find a direct correlation between anxiety and the number of advantageous choices in the rIGT in the current study, the correlation that emerged between %LG and rIGT performance was in fact inversed to that found between %LG and anxiety in both males and females, which indirectly supports a negative relationship between anxiety and decision-making.

Both male and female animals learned to differentiate the long-term advantageous arm from the long-term disadvantageous arm and the empty arm as the task progressed. Previous studies in rats [29], [36], [47], mice [29] and humans [41], [45], [47], [48], [49], [50] have shown clear differences in IGT performance between sexes. It should be noted that although on average IGT scores between males and females differ, both in humans [48] and rats (Van den Bos, unpublished observations), a strong overlap exists between the IGT scores of the two sexes, so that differences may not appear unless group sizes are large. Moreover, male Wistar rats outperform the currently used male Long-Evans rats [29], so that strain differences could contribute to the difference between the current and earlier studies.

### Neurobiological substrate

Performance in the rIGT depends on frontostriatal functioning [30], [34], [35]. This prompted us to study frontostriatal activity in rats receiving different amounts of maternal care, using the immediate early gene c-fos as a marker. The areas that were selected are related to reward, punishment and motivation and implicated in IGT performance, both in humans and in rats [51], [52], [53], [54], [55], [56], [57], [58], [59], [60], [61], [62], [63], [64], [65], see [30] for review.

Strong correlations were observed between c-fos activity in the mPFC (prelimbic cortex and infralimbic cortex) and rIGT decision-making performance. These observations correspond to earlier findings in (male) Wistar rats not characterized for LG received early in life [30]. Both in the earlier and the current study, good rIGT performance was associated with *decreased* c-fos expression in the mPFC. Which population of neurons is responsible for the changes in c-fos expression cannot be discerned from these investigations, but indirect evidence points to a role of GABAergic interneurons. Thus, transient mPFC inactivation by muscimol (GABA-A agonist) or baclofen (GABA-B agonist) resulted in poorer IGT performance as well as higher anxiety levels [34].

A growing body of literature shows that the mPFC is critically involved in strategy shifting, behavioral flexibility and goal-directed learning behavior by encoding task-rules [66], [67], [68], [69], [70], [71], [72], [73]. Functional integrity of the mPFC may allow for the coupling of the history of the animal's choices and rewards as well as behavioral flexibility, to generate and implement an optimal decision-making strategy under conditions of uncertainty. Involvement of the mPFC in the human IGT has been especially associated with punishment processing [74] and risk anticipation [75]. In this scenario, the mPFC contributes to cognitive control over emotional influences on behavior, allowing the subject to maintain a long-term perspective and withhold responding to immediate rewards or losses [76], [77]. Interestingly, the correlation between rIGT performance and c-fos activity in the mPFC did not depend on the LG background, i.e. when data were corrected for the amount of LG that pups received the correlations still existed, and actually became stronger. This was also observed for the cingulate area, which in an earlier study [30] did not correlate with rIGT performance.

Conversely, correlations between rIGT performance and c-fos expression in other areas (lateral orbitofrontal cortex; nucleus accumbens shell) disappeared or became just a trend (insular cortex) when correcting for the amount of LG. This suggests that the rIGT-related activity in the lateral OFC, nucleus accumbens shell and insular cortex might critically depend on important factors in the early life environment such as LG. Recently, several studies have clearly indicated a role for the OFC in rodent versions of IGT decision-making tasks [64], [65], [78]. The degree of OFC involvement may depend on the level of ambiguity experienced by the subject, i.e. the OFC may be important when individuals are learning the reinforcement contingencies and ambiguity is high [79]. Once uncertainty is reduced, the OFC may play less of a role in maintaining the optimal choice strategy. Indeed, the OFC has been suggested to play a role in integrating potentially salient information about environmental contingencies [80], and to use this information to assign a value to a reward and signal outcome expectancies, thus influencing action selection [81], [82], [83], [84]. If so, (female) rats receiving little LG early in life may be less able to assess the expected values of the different options in the initial stages of the task. This agrees with the fact that they were still in the exploratory phase after 120 trials.

The insular cortex is involved in anticipation and receipt of risky, aversive stimuli, such as switches in a choice task [85], [86], [87]. In humans, damage to the insula is associated with a decrease in sensitivity to differences in expected value between choice options and with a concurrent decrease in the amount of risky choices they make [88]. In line with this, increased insula activation during risky decision-making was reported after choosing from a disadvantageous card deck and has been associated with switching choices after a loss as well as with harm avoidance and neuroticism in healthy humans [62], [89]. Also in rats, the insula has been shown to play a role in the anticipation of reward [90] and the memory of the incentive value of this reward [91]. The insular cortex is thought to be a key structure in integrating representations of the homeostatic state (interoceptive or bodily state) associated with prior experiences and using that to guide future motivated behaviors, including risky decision making [92], [93], [94]. Accordingly, differences in insular cortex activity due to differences in the amount of LG may translate into differences in the perception of risks associated with a choice and hence to differences in rIGT performance. Thus, high LG animals may have a lower insular cortex activity while performing the IGT, and thus are less sensitive to taking risks, which expresses itself as a good IGT performance.

Early life environment, including maternal care, is known to have lasting neuroendocrine consequences, with low amounts of LG generally being associated with impaired negative feedback regulation of the HPA axis, resulting in higher glucocorticoid exposure [19]. Increased glucocorticoid exposure (particularly when taking place over an extended period of time) was reported to change the dendritic complexity of pyramidal cells in the prefrontal cortex [8], [95], [96], [97]. We therefore considered the possibility that the influence of LG on the correlation between c-fos expression and rIGT decision-making was mediated by changes in dendritic complexity. However, our data give insufficient support for this thesis. With regard to morphology we did not observe any relationship between maternal care and dendritic properties in the insular cortex. With regard to infralimbic pyramidal cells, the amount of LG in males correlated negatively with the number of branch points in basal dendrites. In females no significant correlations were observed between the amount of LG and infralimbic basal or apical dendritic morphology, although this might in part be due to the low sample size. Overall, the correlation between rIGT performance and dendritic morphology was complex, with sex-dependent differences. Therefore, we interpreted these data conservatively. While our results suggest that rIGT performance and the associated c-fos activity might depend on structural properties of the principal neurons in the selected areas, we cannot exclude the possibility that other characteristics of these cells or structure/function of neurons other than pyramidal cells contribute to the overall outcome.

In conclusion, our data show that the amount of care received by an individual rat from its mother affects adult decision-making performance as well as the associated c-fos activity particularly in the lateral OFC (in females), insular cortex and nucleus accumbens shell. It is tempting to speculate that early life environment may impact on the development of these parts of the brain such that their functionality is lastingly altered. If so, this may bear relevance to the fact that many neuropsychiatric disorders that are associated with early life adversity, such as mood disorders, anxiety disorders and schizophrenia, involve aberrant function of the frontostriatal circuit (for reviews see [24], [98]).
